# Transformation of 1D/2D High‐Surface‐Area Hierarchical Titanium Sulfate Structures to Stable, Morphology‐Preserving Titania with Tailored Properties

**DOI:** 10.1002/smtd.202500168

**Published:** 2025-07-01

**Authors:** Katelyn Sowards, J. Reveles, Hector Medina

**Affiliations:** ^1^ School of Engineering Liberty University 1971 University Blvd Lynchburg VA 24515 USA; ^2^ John Hopkins Center for Talented Youth 5801 Smith Ave #400 Baltimore MD 21209 USA

**Keywords:** 2D material synthesis, hierarchical materials, tailored nanomaterial properties, titanium dioxide photocatalyst

## Abstract

This report outlines a novel, facile process for the transformation of hierarchical enhanced surface area structures (HESAS) of titanium sulfate into titania. The transformation process preserves the HESAS morphology while providing tunable enhanced properties, based on the phase and degree of transformation. To demonstrate our process, a controlled thermo‐chemical transformation strategy is implemented using four maximum temperatures (650, 750, 850, and 950 °C) in natural air or argon‐rich environments, under various heating rates, and for two types of precursor HESAS. The resulting titania HESAS are characterized using scanning electron microscopy (SEM), energy dispersive spectroscopy (EDS), and thin‐film X‐ray diffraction (XRD). Furthermore, both ab initio and semi‐empirical quantum mechanics computational studies are conducted to provide insights into the diffusion mechanisms involved and the associated energetics. The transformed materials exhibit retention of the hierarchical features from the precursor HESAS. Furthermore, the degree of anatase or rutile formed is controlled based on the thermal kinetics of the process. Computational studies show that SO_3_ release is the main mechanism underlying the transformation, with the removal energy barrier increasing with the number of SO_3_ released. This work reveals a pathway for a scalable, low‐cost manufacturing process for the design and fabrication of advanced titania‐based photocatalytic materials with tailored properties.

## Graphical abstract

This report outlines the transformation of hierarchical titanium sulfate structures to titanium dioxide. During the transformation process, the desirable HESAS morphology is maintained. The maximum conversion temperature is adjusted to tailor the phases present in the final product. The thermo‐chemical transformation process is successful with various titanium sulfate precursor morphologies.

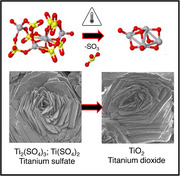

## 1 Introduction

Surfaces define the boundary between materials and their environment; therefore, surface characteristics are critically important for controlling and predicting the behavior of materials. The functionality of a given material can be improved by designing it with a structure that enhances surface area. Hierarchical enhanced surface area structures (HESAS) maximize the distinct and valuable attributes of surfaces. HESAS are comprised of nano/micro‐scale building blocks assembled to produce micro/meso‐scale structures that increase surface area. The architecture of HESAS can be manipulated to optimize performance in a variety of applications.

**Figure 1:**
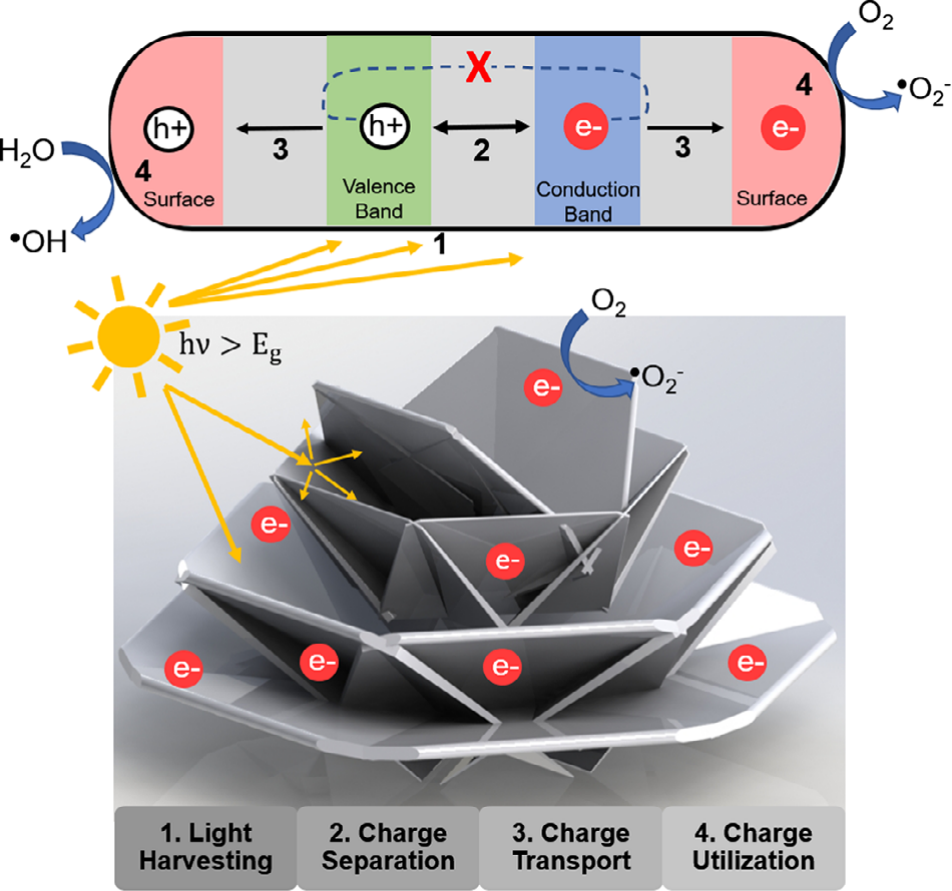
Photocatalytic reaction hosted on a hypothetical HESAS structure. The top of the figure showcases the charge separation, transport, and utilization as it occurs in the host material. The bottom of the figure exhibits how HESAS can enhance each step in the photocatalytic reaction. (Source:^[^ ^22^ ^]^).

Hierarchical enhanced surface area structures improve the functionality of materials in applications such as water purification,^[^ ^1^, ^2^ ^]^ photocatalytic degradation of pollutants,^[^ ^3^, ^4^, ^5^, ^6^ ^]^ photocytotoxicity (e.g., photokilling of malignant cells),^[^ ^7^, ^8^ ^]^ energy generation (e.g., solar cells),^[^ ^9^ ^]^ energy storage (e.g., Li‐ion batteries),^[^ ^10^ ^]^ and sensors.^[^ ^11^ ^]^ Many of these applications rely on photocatalytic materials, but the organization of these materials into HESAS can significantly enhance their photocatalytic performance by improving each step in the process: light harvesting, charge separation, charge transport, and charge utilization. **Figure** **1** provides a visual overview of the photocatalytic process. Light harvesting is improved by providing more surface area for incident EMR interactions.^[^ ^12^, ^13^ ^]^ Surface states create sites for trapping electrons and holes, which can help reduce the recombination rate, thus improving charge separation.^[^ ^14^ ^]^ Charges transport through the material structure. Similar to macro‐electric phenomena, charge transport through the structure will be dictated in part by its geometry. The increased surface area provides more opportunities for interaction between the material and the targeted species, which enhances charge utilization. Aside from photocatalytic applications, HESAS could be highly useful in the development of micro‐mixers,^[^ ^15^, ^16^ ^]^ chemical catalysts,^[^ ^17^, ^18^, ^19^ ^]^ and super‐hydrophobic surfaces.^[^ ^20^, ^21^ ^]^ The authors previously published an application‐focused review of titanium dioxide HESAS.^[^ ^22^ ^]^

Particularly, titanium dioxide HESAS have exhibited significant potential to increase performance in applications such as solar cells,^[^ ^23^, ^24^ ^]^ self‐cleaning and antibacterial surfaces,^[^ ^25^, ^26^ ^]^ water purification devices,^[^ ^27^ ^]^ lithium battery anodes,^[^ ^28^, ^29^ ^]^ degradation of pollutants,^[^ ^5^, ^30^, ^31^ ^]^ and sensors.^[^ ^32^, ^33^, ^34^ ^]^ Titanium dioxide, as a material alone, has many beneficial intrinsic attributes. For example, as a semiconductor, titanium dioxide has a bandgap that can be tuned to induce desired photocatalytic reactions in the visible and near‐visible region. Titanium dioxide is a transition metal oxide with multiple polymorphs, rutile and anatase being the most common. Titanium dioxide in standard morphologies is easy to obtain and generally affordable. It is widely used as a white pigment for paints, cosmetics, and food products. When titanium dioxide is formed into a HESAS, the intrinsic benefits of the material are maximized. However, methods for the fabrication of titanium dioxide HESAS can be complex and difficult to expand beyond laboratory usage, such as template‐assisted methods^[^ ^35^ ^]^ or sol‐gel methods.^[^ ^36^, ^37^, ^38^ ^]^

We have previously reported the synthesis of a titanium‐and‐sulfur‐based HESAS with repeatable rosette‐like morphologies.^[^ ^39^, ^40^ ^]^ While this synthesis method is straightforward and can yield a significant quantity of HESAS with a low barrier to entry, the titanium sulfate chemistry of this HESAS inherently introduces challenges to broader applicability. First, titanium sulfates are not semiconducting materials; they are not practically suitable as photocatalysts. Second, titanium sulfates are known to be hygroscopic. In aqueous solutions or humid environments, they can hydrolyze, thereby limiting their application. In contrast, titanium dioxide is highly useful as a photocatalyst, stable, inert, insoluble in water, and generally considered safe for humans and the environment.

The structural characteristics of the titanium sulfate HESAS were highly desirable, but for the aforementioned reasons, their chemical composition was a limiting factor for broader adoption. As a result, we sought a method to transform titanium sulfate HESAS into titanium dioxide HESAS without losing the beneficial structural features produced in the original synthesis process. In this report, we outline our success in developing a method to convert titanium sulfate HESAS to titanium dioxide HESAS while maintaining original structural features. This method is facile, scalable, and relies on a controlled thermo‐chemical transformation process after HESAS synthesis. Additionally, ab initio and semi‐empirical quantum mechanics computational studies are reported to provide insights into the diffusion mechanisms and the associated energetics involved in the thermochemical conversion process. Our findings contribute to the development of a titanium dioxide HESAS synthesis process that is straightforward and scalable for industrial application. Synthesizing titanium dioxide as a HESAS functionalizes the material for better performance and broadens the potential adoption of HESAS for various applications.

## 2 Results and Discussion

### 2.1 Structural Characteristics of Converted HESAS

**Figure 2:**
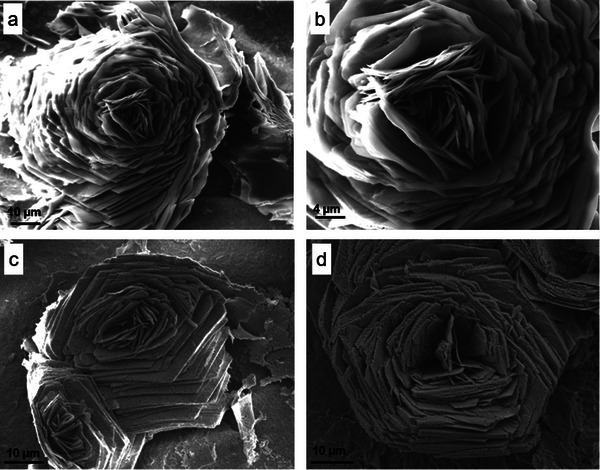
Rosette‐like HESAS after thermo‐chemical processing, converting the structure to titania. a,b) First successful HESAS conversion from titanium sulfate to titanium dioxide, shown at differing magnifications. The specimen was treated to 850 °C. The flower‐like structure retained its characteristic thin petals and circular arrangement. c) Another rosette‐like HESAS formation imaged after conversion to titanium dioxide via treatment at 850 °C. d) Rosette‐like HESAS transformed to titanium dioxide chemistry after treatment at 650 °C.

Upon examination with SEM, it was evident that the converted formations retained their structural characteristics when compared to the titanium sulfate samples prior to conversion. **Figure** **2** showcases HESAS after conversion. The lamellar structure of the rosette‐like formations was still evident in the converted samples. Based on a review of SEM images, the thickness of the individual petals was in family with the titanium sulfate samples, albeit the texture of the petal surfaces became slightly rougher, somewhat similar to a sandblasted surface. This textural change may be indicative of the formation of rutile titanium dioxide on the surface of the structure. Based on our maximum temperature of 850 °C, it is feasible that rutile would have formed on the otherwise primarily anatase structure. Additional discussion is provided in subsequent sections regarding the difference in surface texture correlated with maximum hold temperature.

**Figure 3:**
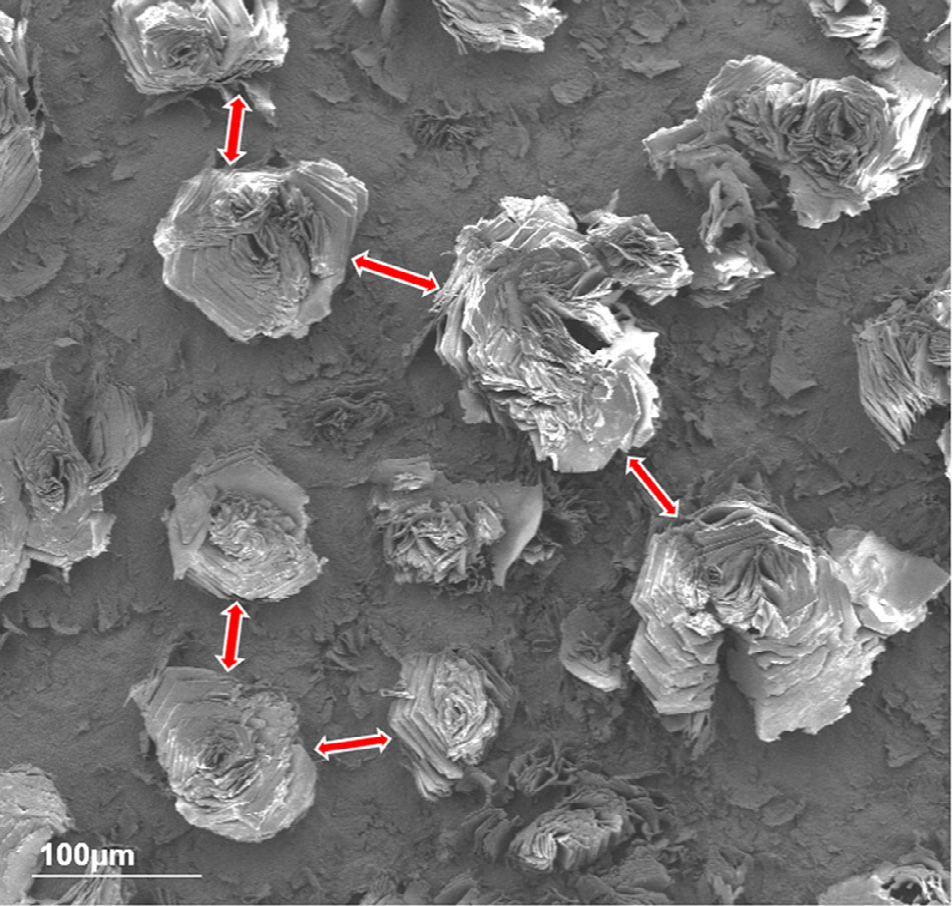
Separations were identified in the overall coating structure after the conversion process. Separations between structures are identified with red arrows.

When viewed at lower magnification, cracking was evident across the surface, as shown in **Figure** **3**. The transformation process created natural separations in the landscape of rosette formations. These cracks were initially assumed to be indicative of shrinkage in the structure. The overall phase change from titanium sulfate to titanium dioxide results in lattice structural changes. These lattice structure changes could cause volume changes, which, if not uniformly accommodated, could cause cracking. However, dimensional investigation did not corroborate large changes in structural feature size as a result of the thermo‐chemical conversion process. While shrinkage may be a minor contributing factor to the cracking phenomenon, it is also probable that the cracking could be caused by rapid reorganization in the crystal structure via the exit of water molecules and/or sulfur oxides. Temperature change at non‐optimum rates may contribute to thermal shock and excessive expansion/contraction of the structures.

### 2.2 Chemical Characteristics of Converted HESAS

After structural examination, the converted samples were evaluated under EDS. The EDS evaluation provided no evidence of sulfur in the converted samples. For all rosettes measured, the average EDS readings were 31.8 at% Titanium and 67.4 at% Oxygen. The EDS data collected aligns closely with the atomic percentages expected for titanium dioxide. The data was collected on multiple individual structures on each coupon and across multiple distinct coupons. Additionally, due to this clear transformation to titanium dioxide, charging effects were more pronounced when collecting SEM images, which may be noted when comparing the titanium sulfate and titanium dioxide formation images.

On average, EDS data of the standard samples before thermo‐chemical treatment exhibit approximately 76% Oxygen, 14% Sulfur, and 8% Titanium in atomic percentage. (EDS cannot account for hydrated species, as it does not collect data for hydrogen.) With the available data and reaction conditions, there was no evidence to disprove that the structure is a titanium sulfate, possibly also containing partially hydrated species. Titanium sulfate and its hydrated forms exhibit higher sulfur composition than titanium. Titanyl sulfate exhibits a near equivalent atomic percentage of titanium and sulfur. In all samples, a higher atomic percentage of sulfur was always observed. Additionally, as previously reported,^[^ ^39^ ^]^ the removal of the oxide layer is key to the formation process. This indicates that additional free titanium ions are necessary for the formation. Titanium (III) ions are not present until the bare titanium begins to be etched by the sulfuric acid; when reacting with the protective titanium dioxide layer, titanium (IV) ions are the primary product. Titanium (III) ions are often indicated by a purple/violet color transformation,^[^ ^41^ ^]^ and the precipitate for the standard rosettes before conversion is lightly colored purple.

Many synthesis parameters in the chosen system have been shown to affect the resulting HESAS. For example, environmental humidity must be controlled to produce the rosette‐like HESAS. Modifying acid molarity in the growth solution has caused changes in the formations. Additionally, changes to acid etching pretreatment conditions (time, temperature, and molarity) as well as alternate acids for etching pretreatment have been investigated. Correlations between these parameters and their effect on surface roughness are ongoing. Substrate surface roughness may be a key parameter in controlling HESAS formation. Therefore, functionalization of materials via conversion to HESAS structure can be controlled by synthesis parameters. While the focus of this report is on the chemical conversion of the structure, future reports will outline our findings regarding how synthesis parameters impact HESAS formation. Both the chemistry and the surface structure are critical features to control the functionalization of titanium dioxide for a chosen application.

### 2.3 Thermo‐Chemical Conversion Process Development

In 1958, Sullivan and Cole^[^ ^42^ ^]^ reported the preparation of colloidal titanium dioxide via annealing of a hydrous titanium dioxide compound prepared via titanium sulfate solution. Based on differential thermal analysis, loss of water from the structure was found to occur ≈150 °C, loss of sulfur trioxide ≈650 °C, crystallite and particle growth at 600 °C, and transformation from anatase to rutile between 700 and 900 °C. This study was the most similar in nature to the rosette‐like HESAS precursor. No other studies were found pertaining to the transformation of nanostructured titanium sulfate, though a study was found pertaining to titanyl sulfate transformation.^[^ ^43^ ^]^ Based on this limited information, our thermo‐chemical conversion process was developed. The successful process to convert the titanium sulfate structures to titanium dioxide consisted of three segments. The first segment brought the samples to ≈200 °C, and the primary purpose of the segment was to remove trapped water molecules in the compound. The second segment brought the samples to ≈450 °C; this segment was added to allow ample time for the decomposition process. The third segment ensured a full conversion to titanium dioxide.

### 2.4 Computational Studies of SO_3_ Removal Mechanism in Thermo‐Chemical Conversion Process

The exact mechanisms of the thermal decomposition of Ti(SO_4_)_2_ into TiO_2_ have not been completely elucidated. However, it could comprise the breaking down of sulfate groups at elevated temperatures, releasing sulfur trioxide, SO_3_, similar to the mechanisms observed in TiOSO_4_,^[^ ^44^ ^]^ and further generalized for various transition metal sulfates.^[^ ^45^ ^]^ Based on those theories, when Ti(SO_4_)_2_ is heated, the energy supplied breaks the bonds in the sulfate groups, causing the sulfur oxides SO_3_ to volatilize and leave behind a titanium‐containing residue, similar to the observed for the thermal decomposition of some transition metal sulfates M(SO_4_) with M = Fe, Co, Ni, Cu, and Zn.^[^ ^45^ ^]^ The general reaction for the process investigated in this paper can be summarized as Ti(SO_4_)_2_→TiO_2_ + 2SO_3_.

As temperature increases, titanium atoms in the residue bond with oxygen to form titanium dioxide, a stable and thermodynamically favored product, similar to the reported case of spherical TiO_2_ powders by thermal hydrolysis of Ti(SO_4_)_2_.^[^ ^46^ ^]^ As our experiments show, complete decomposition typically occurs at high temperatures when all volatile sulfur species are driven off, leaving behind solid titanium dioxide. The exact decomposition pathway can vary based on conditions like temperature and pressure, and we modeled it as a series of stepwise eliminations involving removal energies that are overcome by the system's thermal energy. Notably, as demonstrated in this investigation, the initial structure of the titanium atoms in Ti(SO_4_)_2_ is generally retained, generating a unique configuration of the titanium dioxide product.

**Figure 4:**
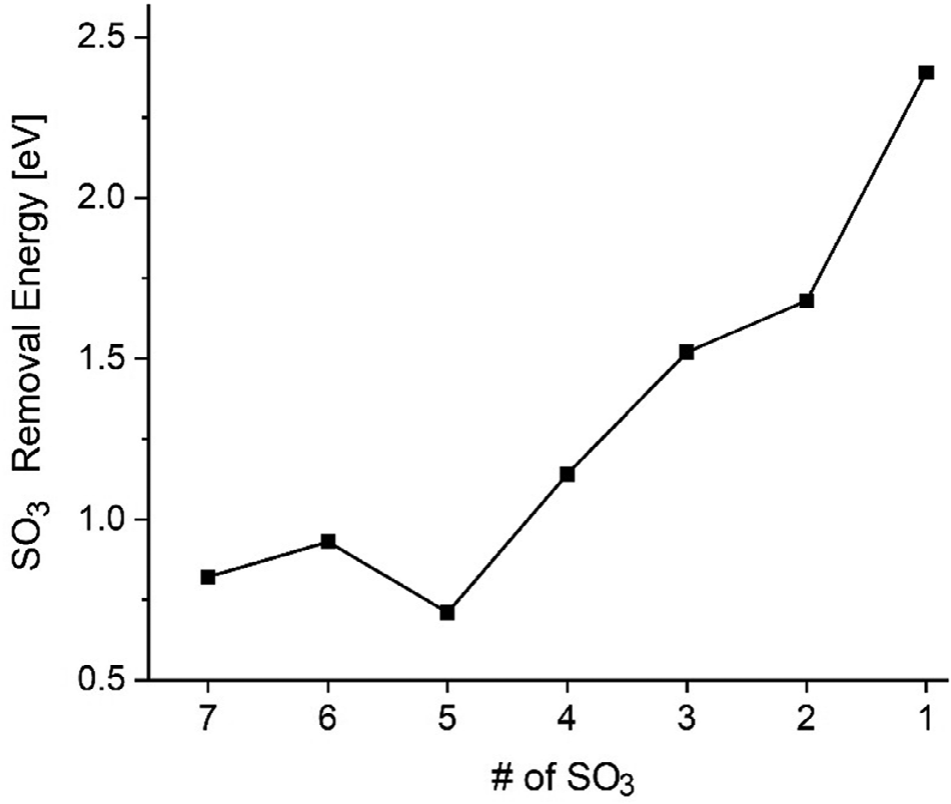
SO_3_ removal energies (eV) for the Ti_4_O_8_(SO_3_)_n_ cluster model indicate a general trend of increasing energy barrier as the number of released SO_3_ increases.

**Figure 5:**
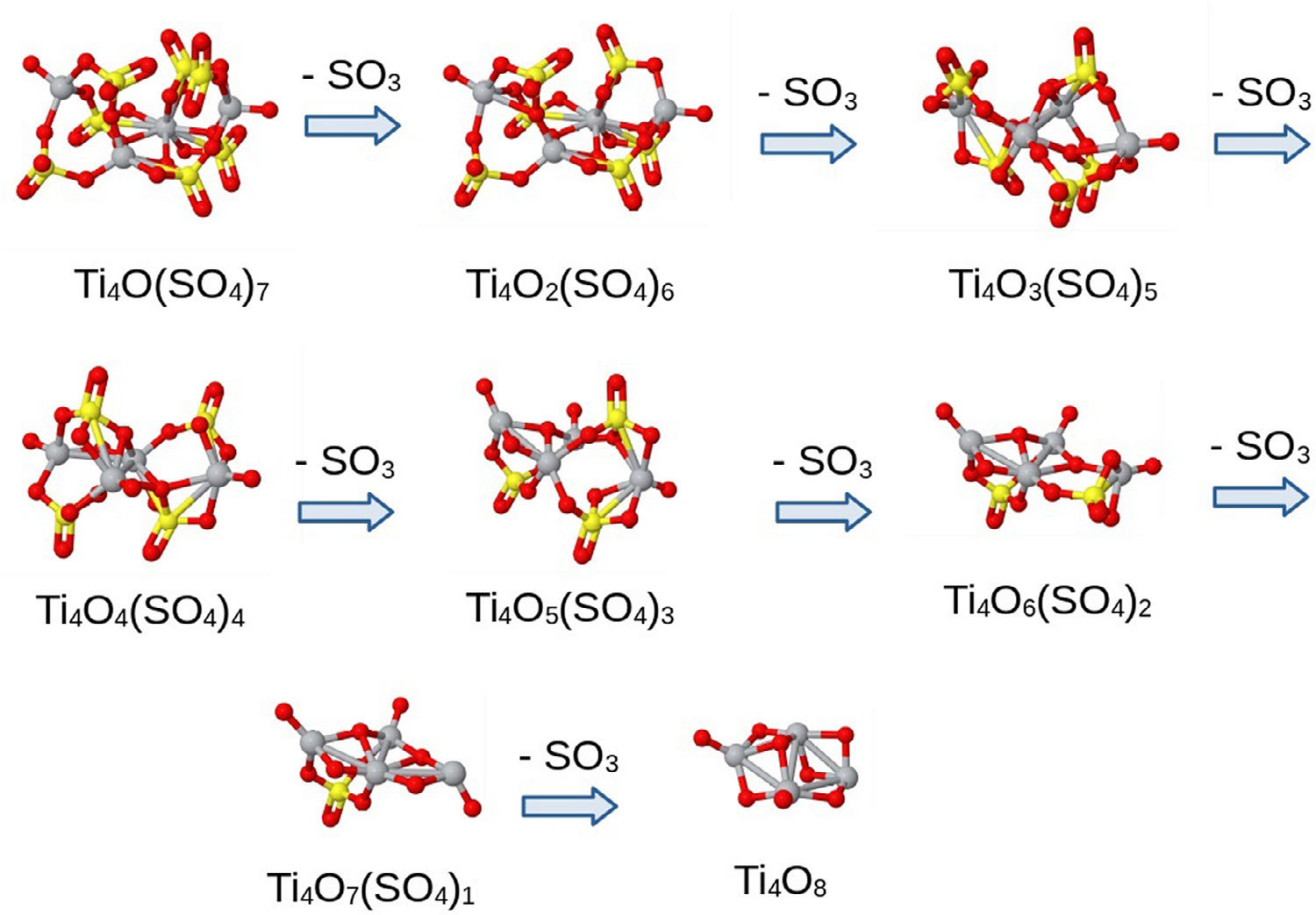
Optimized geometries for the Ti_4_O_8_(SO_3_)_n_ cluster model with *n* = 0 to 7. Ti, S, and O atoms are gray, yellow, and red circles, respectively.

We performed a series of successive geometry optimizations to calculate the relaxed geometries after each SO_3_ release and the corresponding energy required for each step. Notably, our proposed model was energetically unstable, releasing the first SO_3_ molecule during its initial optimization. Therefore, our results are presented starting from the Ti_4_O(SO_4_)_7_ cluster model, chosen for its relevance in modeling the decomposition process. The SO_3_ removal energy for each step is calculated using the equation:

(1) $$RE_{n}=\;E\left(Ti_{4}O_{8}{\left(SO_{3}\right)}_{n}\right)-E\left(Ti_{4}O_{8}{\left(SO_{3}\right)}_{n-1}\right)-E\left(SO_{3}\right)$$

where E(Ti_4_O_8_(SO_3_)_n_), E(Ti_4_O_8_(SO_3_)_n−1_), and E(SO_3_) are the total energies of the optimized Ti_4_O_8_(SO_3_)_n_, Ti_4_O_8_(SO_3_)_n−1_, and SO_3_ clusters, respectively. **Figure** **4** shows the removal energies, ranging from 0.82 to 2.84 eV, with a general increasing trend, indicating a higher energy barrier, in general, for successive SO_3_ releases. **Figure** **5** displays the optimized geometries for each cluster, illustrating the structural changes at the atomic level as SO_3_ groups are removed, noting the ultimate titania cluster (TiO_2_)_4_ structure, yet with chemical properties relevant to the titanium dioxide product.

All geometry optimizations were performed without symmetry constraints, allowing each Ti₄O_x_(SO₄)₈₋_x_ cluster to relax into its actual minimum‐energy configuration. Interestingly, the most stable structures often turn out to be highly symmetric and geometrically compact, maximizing the number of Ti–O–S bonds to each SO₃ unit. Such high‐coordinate binding of a SO₃ fragment (analogous to a chelating, multidentate ligand) greatly enhances its attachment energy.^[^ ^47^ ^]^ In other words, when a SO₃ group bridges multiple Ti centers in a rigid, symmetric cluster, removing it necessitates breaking several metal‐oxygen bonds, costing significantly more energy, which explains the non‐monotonic trend observed in the SO₃ detachment energies (as seen in Figure 5). The removal energy is determined by the number of coordination bonds that must be cleaved. More symmetric (hence more compact and rigid) clusters have SO₃ units locked in place by multiple linkages and exhibit higher SO₃ removal energies.^[^ ^48^ ^]^ Conversely, less symmetric, more open structures present SO₃ groups that are bonded in fewer places and are more easily removed. This correlation between point‐group symmetry/geometric rigidity and ligand detachment energetics is consistent with known trends in metal cluster chemistry, wherein highly symmetric clusters (e.g., those tending toward icosahedral or polyhedral frameworks) achieve extra stability from maximized bonding interactions.^[^ ^49^ ^]^ All the Ti₄_x_(SO₄)₈₋_x_ clusters considered here are closed‐shell species, so these energetic trends primarily reflect structural coordination effects rather than electronic shell effects.

It is interpreted that the idealized static states shown in Figure 5 are achieved at a temperature near 700 °C, where the system's thermal energy overcomes the energetic barriers shown in Figure 4. The calculated removal energy barriers are for a finite system, which inherently possesses more structural flexibility than a bulk material; therefore, the phase transformation temperature could be lower than 700 °C. The results provide crucial insights into the atomic‐level mechanisms driving SO_3_ release and titanium dioxide formation from titanium sulfate. Interestingly, the Ti_4_ cluster retained its configuration throughout the process, supporting our conclusion that the thermal removal of SO_3_ can keep some of the structural geometries of the parent titanium sulfate.

### 2.5 Alternate Precursor Structures Converted

**Figure 6:**
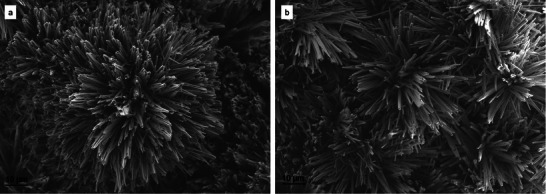
a) Titanium sulfate dandelion‐like HESAS that has not undergone conversion. b) Another dandelion‐like HESAS after thermo‐chemical conversion process to titanium dioxide. The images are taken from the same sample but from differing locations.

Aside from the standard rosette‐like structures, which we have previously reported on,^[^ ^39^, ^40^ ^]^ the thermo‐chemical conversion method was also tested on dandelion‐like structures. After thermo‐chemical treatment, the dandelion‐like formations retained their overall structure as shown in **Figure** **6**, but their chemical makeup, when examined by EDS, was indicative of titanium dioxide. This finding exhibits the applicability of this conversion process on a broad range of titanium sulfate structures. Most critically, both formation geometries have retained their desirable structural features, indicating a straightforward method for the fabrication of hierarchical enhanced surface area structures of titanium dioxide. The origin of the dandelion‐like structure is an open research question, as these were found after a period in storage. The dandelion‐like structure was originally fabricated in the same manner as the rosette‐like structures and exhibited a similar titanium sulfate composition.

### 2.6 Varying Maximum Temperature During Conversion

The initial thermo‐chemical conversion process relied on 850 °C as the maximum temperature. This temperature was chosen to ensure the titanium sulfate structures would fully convert to titanium dioxide. However, based on the literature and our computational results, we believed the full conversion could take place at a lower maximum temperature. By exploring alternate temperatures, we hypothesized that we could control the phases present in the final converted structure and possibly mitigate the cracking observed in the overall coating.

The alternate thermo‐chemical conversion methods relied on the same heating ramp rates as the method previously described. However, each test adjusted the maximum temperature to which the structure was heated. Four different maximum temperatures were assessed; these were 650, 750, 850 (standard method), and 950 °C. The alternate temperatures were tested in the same oven with standard atmospheric gases and ambient pressure. Overall, it was challenging to quantify any change in overall coating coverage based on the heat treatment method. All samples still exhibited some cracking and separation compared to the initial titanium sulfate HESAS coating. The geometry of the titanium sulfate rosettes prior to heat treatment had a more substantial effect on the amount of cracking observed.

When reviewing individual rosette‐like HESAS formations, there was a correlation between the porosity of the “petals” and the temperature to which the structure was treated. Each titania rosette on all sample types exhibited porosity on the thin petal edge. Considering this is the direction of growth, the energetic unfavourability of this surface and its susceptibility to degradation are anticipated. The high‐energy petal edge is a prime location for significant adsorption and desorption activity. Based on our porosity observations, desorption must occur at a higher rate in this location.

**Figure 7:**
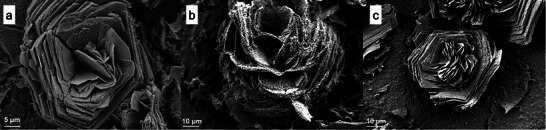
Rosette‐like HESAS exhibiting porosity after conversion from titanium sulfate precursors to titanium dioxide. a) Samples were treated to 650 °C. Porosity can be observed on petal edges. b) Sample treated to 750 °C and exhibits significant porosity over the entire structure. The pores are less than 1 µm in diameter. C) Sample treated to 850 °C exhibits roughness on the petal faces but significantly less porosity than the sample treated to 750 °C.

The porosity of the petal faces changed substantially with the maximum temperature of the treatment, as exhibited in **Figure** **7**. Specifically, limited porosity on the petal faces was observed for the samples treated to 650 °C. However, for the samples treated to 750 °C, the petal faces exhibit substantial porosity, with pores less than 1 µm in diameter. Samples treated to 850 °C did not exhibit much porosity, though the petal surfaces did exhibit a rougher texture when compared to the titanium sulfate precursor. This finding exhibits another useful feature of the developed conversion process in that it can be used to modify porosity within structures. This introduces another dimension to the already hierarchical nature of the rosettes.

Additionally, a set of samples was heat‐treated to 850 °C in an argon‐rich environment to evaluate effects caused by ambient oxygen during the heat treatment. The specimens are still fully converted to titanium dioxide with EDS and XRD data in family with the samples treated in ambient air. To date, this conversion process has only been tested in standard and inert atmospheres. Future attempts with reducing atmospheric conditions may be of interest to evaluate if nonstoichiometric titania could result. Another set of samples was tested in ambient air to 850 °C, but with a much lower heating ramp rate (100 °C hr^−1^) used throughout the process. There was no appreciable difference in chemistry resulting from these structures. Porosity was higher in the samples than in those treated to 850 °C with a faster heating ramp rate (250 °C hr^−1^).

**Figure 8:**
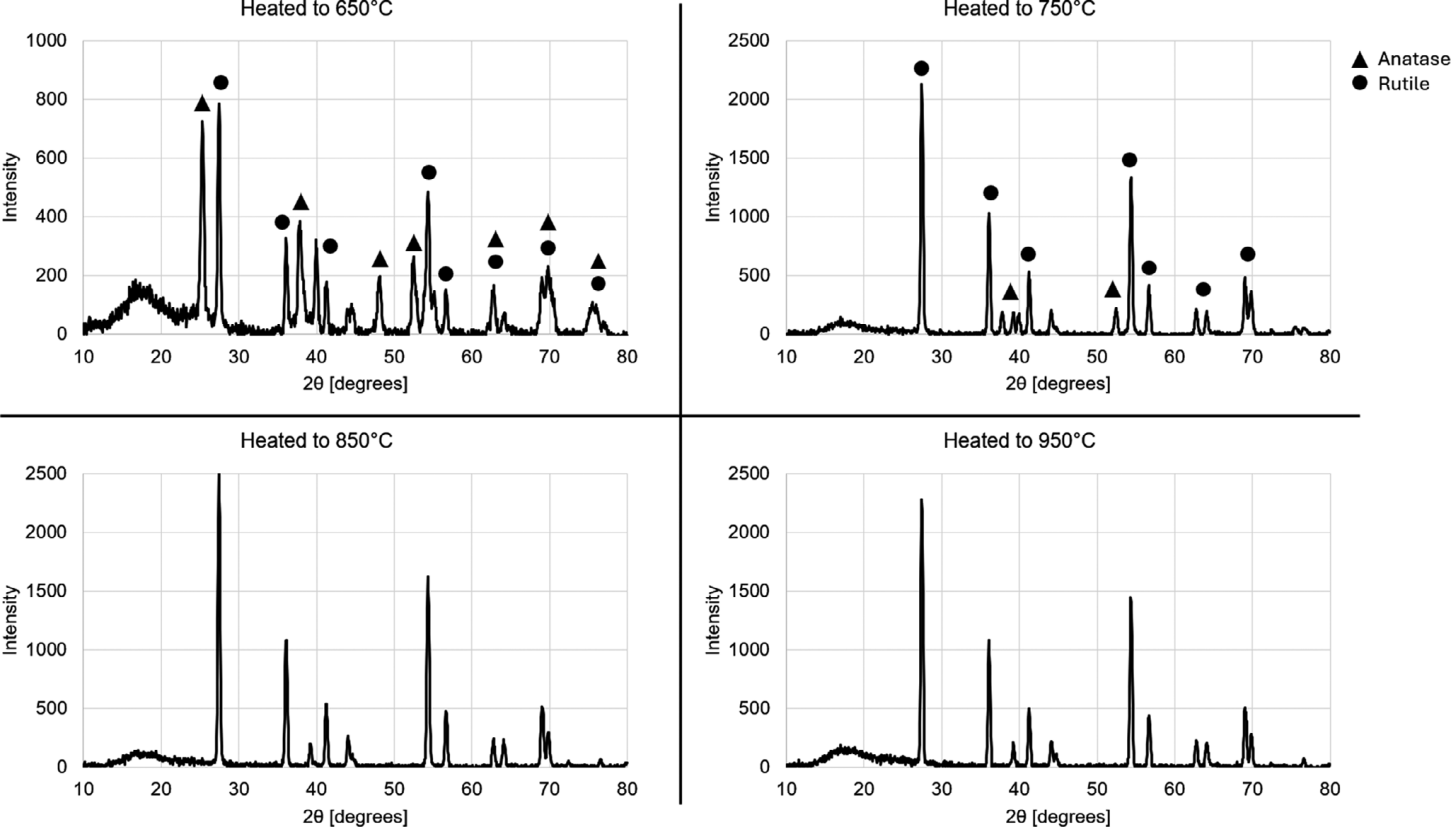
Thin film XRD data of converted samples treated to 650, 750, 850, and 950 °C. The sample treated to 650 °C exhibits a mixed phase of anatase and rutile. The sample treated to 750 °C showcases a substantial but not complete transition to rutile. Anatase peaks are correlated with a triangle; rutile peaks are correlated with a circle. Samples treated to 850 °C and 950 °C exhibit almost identical patterns and are indicative of the rutile phase. Peaks are not marked as they all align with rutile titania.

Each type of sample was examined via thin film XRD to determine the phases present. Upon initial review of the XRD data shown in **Figure** **8**, the sample treated to a max temperature of 650° showed characteristics of a mixed phase of rutile and anatase titanium dioxide. Peaks measured from the sample closely align with characteristic peaks of both rutile and anatase. For the samples treated to 750 °C, the resulting XRD data were much more closely aligned with rutile titanium dioxide, and many of the characteristic anatase peaks were missing from the data. The overall intensity of the peaks measured was also significantly higher than that of the sample treated to 650 °C. For example, the characteristic rutile peak at 27.4° had a relative intensity 3x that of the sample prepared to 650 °C. When reviewing XRD data for the samples treated to 850 and 950 °C, little to no difference could be found upon comparison of their XRD results. Additionally, the results perfectly aligned with the characteristic peaks for rutile titanium dioxide for both samples, indicating this structure had fully transitioned to a rutile phase. Phase quantification based on the XRD was performed via Rietveld refinement using the FullProf engine within Match! Software. The relative weight percentages of each phase were calculated and can be seen in **Table** **1**.

**Table 1:** Phase identification of converted rosette‐like HESAS correlated with thermo‐chemical conversion process maximum temperatures: 650, 750, 850, and 950 °C. Anatase and rutile were the primary phases identified, with only the rutile phase appearing in the samples treated to 850 °C and 950 °C.

Sample	Anatase [wt.%]	Rutile [wt.%]
650 °C	52.9	47.1
750 °C	3.2	96.8
850 °C	0	100
950 °C	0	100

By converting the HESAS to titania, the desirable photocatalytic activity of the material can be leveraged. Anatase has a bandgap of 3.2 eV, and consequently, it requires long‐wavelength UV radiation for excitation. However, rutile has a 3.02 eV bandgap, making it responsive in the visible light region.^[^ ^50^, ^51^ ^]^ Mixed‐phase titania has been shown to increase photocatalytic performance by creating trap states between phases, leveraging the visible light photo‐responsiveness of rutile, and while also relying on the superior photocatalytic behavior of anatase.^[^ ^52^, ^53^, ^54^ ^]^ The exact mechanism of improved charge carrier separation in mixed‐phase titania is still debated. Grain size, structural form, and phase composition are all important factors in the overall photocatalytic activity of mixed‐phase titania. Lei et al.^[^ ^55^ ^]^ have an excellent chapter on the mechanisms and benefits of mixed‐phase TiO_2_ nanomaterials. By careful selection of maximum temperature, this conversion process allows adjustment of the anatase/rutile phase in the final product, which can be used to tailor properties for a targeted application.

## 3 Conclusion and Future Work

This report outlines the successful transformation of titanium sulfate formations to titanium dioxide formations while retaining their beneficial nano‐ and micro‐hierarchical structural features. We have previously reported a straightforward method for the synthesis of these titanium sulfate structures.^[^ ^39^ ^]^ Here we outline a process that facilitates the chemical conversion of titanium sulfate HESAS to titanium dioxide HESAS. This process was successfully tested on two different HESAS geometries (rosette‐like and dandelion‐like). Both geometries retained their beneficial structural characteristics but exhibited no evidence of sulfur in their composition. The porosity and titania phase present in the converted structures can be controlled via careful selection of the maximum temperature. With this conversion process now developed, these titanium sulfate HESAS can be converted to a composition useful for broad application. This methodology retains its low barrier to entry, simple control methods, and therefore, feasibility to scale to industrially relevant applications.

This report investigated the mechanism by which this transformation proceeded via computational methods. To further support the conclusions of that study, thermogravimetric analysis (TG) could be conducted; this method may also be effective at revealing if any other byproducts are produced during this conversion. The SO_3_ gaseous byproducts of this conversion process are believed to be the cause of observed porosity. At higher temperatures, the structure settles into a more favorable state, leading to densification and overall reduction in pore size and quantity. Porosity was an interesting supplemental observation to the primary aims of this paper. To further investigate the evolution of porosity with maximum treatment temperature, BET would be of great benefit. However, techniques for BET analysis of the structures must be carefully developed to ensure measurement accuracy; for highly microporous, chemically reactive, or heterogeneous surfaces, choosing the appropriate adsorbate, pressure window, and degassing conditions is not trivial.^[^ ^56^ ^]^ Poorly designed BET experiments can produce misleading results, and in some cases, the measurement may even perturb the surface being characterized, particularly with N₂‐based analyses that can alter phase behavior or surface roughness in titania systems.

The present study relied on EDS and XRD analysis to evaluate composition changes. These tools are effective for the immediate aims of this report, uncovering a method to transform and stabilize the titanium sulfate structure while maintaining morphology. In the future, XPS analysis could be useful for understanding any trace elements in the near‐surface region, which may be particularly beneficial for those researchers using this technique for catalysts. Recently, Badr et al.^[^ ^57^ ^]^ published a report indicating the role of 1DL metastable titanium dioxide intermediate phase in the creation of unique morphologies. While the synthesis conditions differ in that report from ours, inquiry into whether or not any nonstandard titania phases play a role in this conversion process could be of benefit; Raman spectroscopy could be an effective tool to address this question. Magnéli phase titania is a substoichiometric titanium oxide produced via heat treatment in a reducing atmosphere. Magnéli phases have exceptional utility in electrochemical applications and enhanced electrical conductivity.^[^ ^58^ ^]^ Conducting the outlined conversion process under a reducing atmosphere may further enhance the properties via the phases achieved; this is a subject of future work.

The synthesis method utilized in this report relied on the substrate surface for nucleation and growth. From initial investigations, the structures can be mechanically removed from the surface; however, additional research should be conducted to determine what method(s) are most effective at removing the structures while maintaining their morphology, for applications that require the HESAS without the host titanium substrate. Additionally, the authors acknowledge the potential challenges associated with sulfur‐oxide‐based waste. Any technology (process or product) maturation from laboratory to industrial scale (or technology readiness level) is not without challenges, but in this case, there are existing technologies that could remedy this challenge (e.g., scrubbers). The assessment provided in this report regarding the accessibility and straightforward nature of this synthesis process still stands.

## 4 Experimental Section

### Experimental Methods

To synthesize the titanium sulfate rosettes, coupons of grade 2 titanium were etched in 3 µL of 18 molar sulfuric acid for 30 minutes at 60 °C. After oxide layer removal due to etching treatment, the coupons were rinsed in deionized water until they exhibited a neutral pH. Once rinsed, the samples were dried with lint‐free wipes. Then, the rosette growth solution (RGS) was applied to the surface of the coupon. 18m H_2_SO_4_ was utilized as RGS. The RGS was allowed to evaporate in ambient conditions for 24 h. At this point, the sample surface appeared visibly dry, and upon examination with a scanning electron microscope, standard rosette formations were observed.

After characterizing the structural and chemical features of the standard rosette formations, the coupons were subjected to a thermo‐chemical transformation process under standard atmospheric conditions. The coupons were placed in alumina boats and added to an oven at room temperature. The thermo‐chemical transformation process consisted of three ramp and hold periods. The first ramp heated the coupons to ≈200° and held for 1–2 h. Next, the oven temperature was increased to 450° and held for 1–2 h. The oven was continually heated until it reached 850° where it was held for 3 h to facilitate the complete conversion to crystalline titanium dioxide. After this, the samples were allowed to cool at the maximum rate of the oven. Upon removal of the coupons, the surface of the coupon had transformed to a bright white color, and it was difficult to even see the dark grey titanium substrate.

### Computational Methods

**Figure 9:**
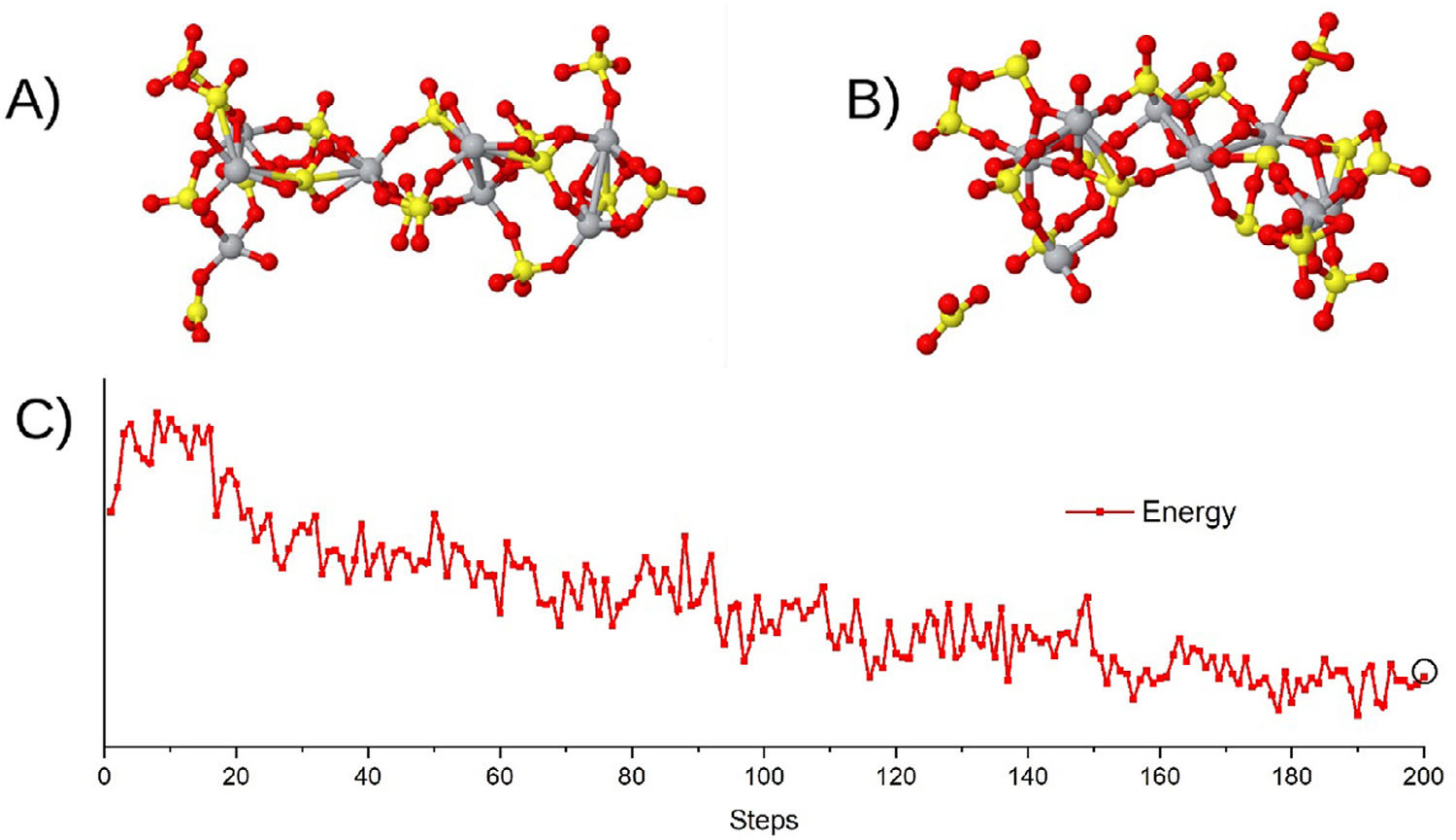
Molecular dynamics simulation of the Ti_8_(SO_4_)_16_ cluster at 700 °C. The system relaxes from the initial geometry (panel A) to the final geometry (panel B), with a released SO_3_, as shown. Panel C shows the simulation's energy profile. The circle point in the energy graph corresponds to the final geometry. Ti, S, and O atoms are gray, yellow, and red circles, respectively.

First, quantum chemical model calculations were performed using the semi‐empirical MO method, MSINDO, which was extensively documented for the first, second, and third‐row main group elements.^[^ ^59^, ^60^ ^]^ The second‐row elements have a (2s, 2p) basis set with different Slater exponents for intra‐ and inter‐atomic integrals comparable to the Pople 6–31G basis set. Inner shells were considered by a pseudopotential after Zerner.^[^ ^61^ ^]^ The combination of reliable structure and energy accuracy, and computation speed for large systems made the semiempirical MSINDO a valuable tool for the current study, which investigated systems up to 88 atoms. A self‐consistent field molecular orbital calculations (SCF) method were carried out with a convergence criterion of an energy change below 10^−5^ Hartrees and NVT Molecular Dynamics (MD) using the Nose‐Hover thermostat at different temperatures (600, 700, 800, and 900 °C) for six ps. The Ti(SO_4_)_2_ was modeled using a cluster, which was a fragment of the crystal structure^[^ ^62^ ^]^ made of eight Ti(SO_4_)_2_ units and with the formula Ti_8_(SO_4_)_16_ shown in **Figure** **9**, panel A. It was found that starting at 700 °C, agreeing with the experimental results, the system releases an SO_3_ unit, as shown in Figure 9, panel B. Also shown, in Figure 9, Panel C, was the simulation's energy profile, where the circle at the rightmost point in the energy graph corresponds to the final geometry.

**Figure 10:**
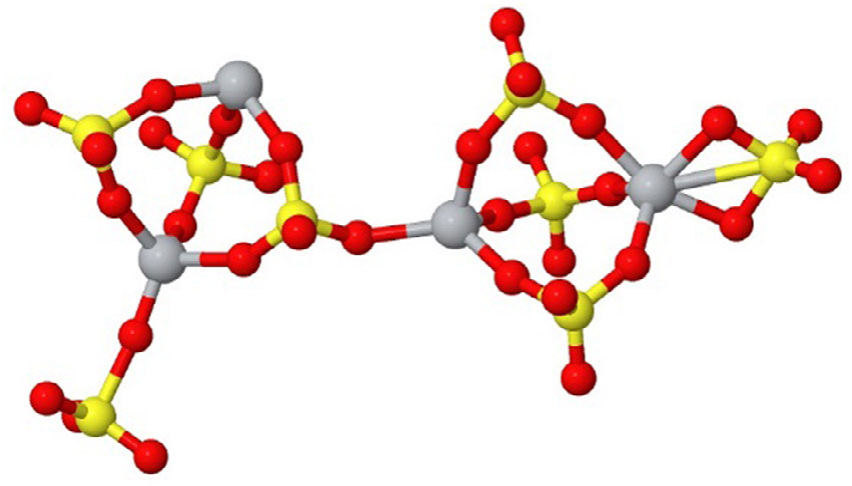
The Ti_4_O_8_(SO_3_)_8_ cluster model. Ti, S, and O atoms are gray, yellow, and red circles, respectively.

Next, the successive SO_3_ thermal release was investigated using an ab initio approach within the Linear Combination of Gaussian Type Orbitals Density Functional Theory (LCGTO−DFT) framework within the Generalized Gradient Approximation (GGA), using the revised gradient corrected PBE96^[^ ^63^ ^]^ and employing the deMon2k software.^[^ ^64^, ^65^ ^]^ All electrons DZVP^[^ ^66^ ^]^ basis sets for Ti, S, and O, combined with the A2 auxiliary function sets, were used. The coulomb potential was evaluated using the variational fitting procedure developed by Dunlap, Connolly, and Sabin.^[^ ^67^ ^]^ For this SO_3_ thermal release investigation, the Ti(SO_4_)_2_ was modeled using a cluster made of four Ti(SO_4_)_2_ units and with the formula Ti_4_O_8_(SO_3_)_8_ shown in **Figure** **10**.

### Ethics Statement

The authors confirm that all experiments and research described in this manuscript were conducted in accordance with ethical standards. No human participants or animals were involved in the research.

## Conflict of Interest

The authors declare no conflict of interest.

## Data Availability

The data that support the findings of this study are available from the corresponding author upon reasonable request.

## References

[1] Z. Ren , Y. Guo , C.‐H. Liu , P.‐X. Gao , Front. Chem. 2013, 1, 18. 24790946 10.3389/fchem.2013.00018 PMC3982538

[2] K. Solanki , S. Sharma , S. Yadav , B. Kaushik , P. Rana , R. Dixit , R. K. Sharma , Small 2023, 19, 2300394. 10.1002/smll.202300394 36950767

[3] J.‐S. Hu , L.‐S. Zhong , W.‐G. Song , L.‐J. Wan , Adv. Mater. 2008, 20, 2977.

[4] R. Gusain , K. Gupta , P. Joshi , O. P. Khatri , Adv. Colloid Interface Sci. 2019, 272, 102009. 31445351 10.1016/j.cis.2019.102009

[5] H. Medina , C. Farmer , Toxics 2024, 12, 610. 39195712 10.3390/toxics12080610 PMC11358922

[6] A. Ware , S. Hess , D. Gligor , S. Numer , J. Gregory , C. Farmer , G. M. Raner , H. E. Medina , Eng. Life Sci. 2024, 24, 202400054. 10.1002/elsc.202400054 PMC11532638 39502856

[7] S. Xiong , Y. Tang , H. S. Ng , X. Zhao , Z. Jiang , Z. Chen , K. W. Ng , S. C. J. Loo , Toxicology 2013, 304, 132. 23295712 10.1016/j.tox.2012.12.015

[8] N. Babayevska , J. Litowczenko , J. K. Wychowaniec , I. Iatsunskyi , M. Jarek , P. Florczak , S. Jurga , Adv. Powder Technol. 2020, 31, 393.

[9] M.‐H. Sun , S.‐Z. Huang , L.‐H. Chen , Y. Li , X.‐Y. Yang , Z.‐Y. Yuan , B.‐L. Su , Chem. Soc. Rev. 2016, 45, 3479. 27255561 10.1039/c6cs00135a

[10] K. Shehzad , Y. Xu , C. Gao , X. Duan , Chem. Soc. Rev. 2016, 45, 5541. 27459895 10.1039/c6cs00218h

[11] S. Bilge , B. Dogan‐Topal , A. Yucel , A. Sinag , S. A. Ozkan , Trends Anal. Chem. 2022, 153, 116638.

[12] X. Li , J. Yu , M. Jaroniec , Chem. Soc. Rev. 2016, 45, 2603. 26963902 10.1039/c5cs00838g

[13] X. Wang , J. C. Yu , C. Ho , Y. Hou , X. Fu , Langmuir 2005, 21, 2552. 15752052 10.1021/la047979c

[14] G. Liu , L. Wang , H. G. Yang , H.‐M. Cheng , G.‐Q. Lu , J. Mater. Chem. 2010, 20, 831.

[15] H. Medina , J. M. Anthony , T. Eldredge , Eng. Res. Express 2023, 5, 015026.

[16] H. Medina , M. Thomas , T. Eldredge , A. Adebanjo , J. Fluids Eng. 2019, 141, 121406.

[17] M. Wang , B. Feng , H. Li , H. Li , Chem 2019, 5, 805.

[18] C. M. A. Parlett , K. Wilson , A. F. Lee , Chem. Soc. Rev. 2013, 42, 3876. 23139061 10.1039/c2cs35378d

[19] C. Zhu , Z. Qi , V. A. Beck , M. Luneau , J. Lattimer , W. Chen , M. A. Worsley , J. Ye , E. B. Duoss , C. M. Spadaccini , C. M. Friend , J. Biener , Sci. Adv. 2018, 4, aas9459. 10.1126/sciadv.aas9459 PMC6118649 30182056

[20] H. Teisala , H.‐J. Butt , Langmuir 2019, 35, 10689. 30463408 10.1021/acs.langmuir.8b03088

[21] S. S. Latthe , C. Terashima , K. Nakata , A. Fujishima , Molecules 2014, 19, 4256. 24714190 10.3390/molecules19044256 PMC6270765

[22] K. Sowards , H. Medina , Appl. Mater. Today 2023, 35, 101962.

[23] W. Gan , H. Niu , X. Shang , R. Zhou , Z. Guo , X. Mao , S. Miao , L. Wan , J. Xu , S. Miao , Physica. Status Solidi A 2016, 213, 994.

[24] J. Liang , G. Zhang , J. Yang , W. Sun , M. Shi , AIP Adv. 2015, 5, 017141.

[25] M. Z. Khan , V. Baheti , J. Militky , J. Wiener , A. Ali , J. Ind. Text. 2020, 50, 543.

[26] A. Wasa , J. G. Land , R. Gorthy , S. Krumdieck , C. Bishop , W. Godsoe , J. A. Heinemann , FEMS Microbiol. Lett. 2021, 368, fnab039. 33864459 10.1093/femsle/fnab039

[27] J. Guo , X. Cai , Y. Li , R. Zhai , S. Zhou , P. Na , Chem. Eng. J. 2013, 221, 342.

[28] K. Yin , Y. Cai , X. Zheng , Z. Deng , B.‐L. Su , H.‐E. Wang , Mater. Lett. 2017, 201, 93.

[29] H. Huang , Z. Yu , W. Zhu , Y. Gan , Y. Xia , X. Tao , W. Zhang , J. Phys. Chem. Solids 2014, 75, 619.

[30] T. X. H. Le , H. Haflich , A. D. Shah , B. P. Chaplin , Environ. Sci. Tehcnol. Lett. 2019, 6, 504.

[31] S. Kwon , M. Fan , A. T. Cooper , H. Yang , Crit. Rev. Environ. Sci. Technol. 2008, 38, 197.

[32] M. M. Zahidi , M. H. Mamat , M. F. Malek , M. K. Yaakob , M. K. Ahmad , S. A. Bakar , A. Mohamed , A. S. R. A. Subki , M. R. Mahmood , Sensors 2022, 22, 5794.35957350

[33] C. Zhao , T. Jing , M. Dong , D. Pan , J. Guo , J. Tian , M. Wu , N. Naik , M. Huang , Z. Guo , Langmuir 2022, 38, 2276. 35138855 10.1021/acs.langmuir.1c02956

[34] Y. Tang , P. Liu , J. Xu , L. Li , L. Yang , X. Liu , S. Liu , Y. Zhou , Sens. Actuators, B 2018, 258, 906.

[35] A. Kaur , B. Bajaj , A. Kaushik , A. Saini , D. Sud , Mater. Sci. Eng., B 2022, 286, 116005.

[36] Y. Ren , G. Zhang , J. Huo , J. Li , J. Alloys Compd. 2022, 902, 163730.

[37] R. Govinidaraj , M. S. Pandian , P. Ramasamy , S. Mukhopadhyay , Bull. Mater. Sci. 2015, 38, 291.

[38] S. Backlund , J.‐H. Smatt , J. B. Rosenholm , M. Linden , J. Dispersion Sci. Technol. 2007, 28, 115.

[39] K. Sowards , H. Medina , Results Surf. Interfaces 2024, 15, 100223.

[40] H. Medina , R. Kohler , Eng. Reports 2020, 2, 12247.

[41] D. Devilliers , M. T. Dinh , E. Mahe , D. Krulic , N. Larabi , N. Fatouros , J. New Mater. Electrochem. Sys. 2006, 9, 221.

[42] W. F. Sullivan , S. S. Cole , J. Am. Ceram. Soc. 1958, 42, 127.

[43] M. Johnsson , P. Pettersson , M. Nygren , Thermochim. Acta 1997, 298, 47.

[44] O. Shaposhnyk , P. Kapustnik , P. Mateychenko , W. Kucharczyk , J. Mater. Res. Technol. 2020, 9, 12201.

[45] H. Tagawa , H. Saijo , Thermochim. Acta 1985, 91, 67.

[46] H. K. Park , Y. T. Moon , C. H. Kim , J. Am. Ceram. Soc 1996, 79, 2727.

[47] C. Liu , X. Zhang , J. Wang , Y. Zhang , Q. Zhao , Y. Li , Y. Li , Adv. Opt. Mater. 2024, 12, 2200214.

[48] L. Chkhartishvili , Molecules 2022, 27, 1469. 35268570 10.3390/molecules27051469 PMC8911741

[49] S. Pande , X. Gong , L. S. Wang , X. C. Zeng , J. Phys. Chem. Lett. 2019, 10, 1820. 30925053 10.1021/acs.jpclett.9b00446

[50] G. Shipra Mital , T. Manoj , Phys. Chem. Rev. 2011, 56, 1639.

[51] A. J. Haider , Z. J. Jameel , I. H. Al‐Hussaini , Energy Procedia 2019, 157, 17.

[52] G. Li , L. Chen , M. E. Graham , K. A. Gray , J. Mol. Catal. A: Chem. 2007, 275, 30.

[53] S. Paul , A. Choudhury , Appl. Nanosci. 2014, 4, 839.

[54] R. G. Nair , S. Paul , S. K. Samdarshi , Sol. Energy Mater. Sol. Cells 2011, 95, 1901.

[55] J. Lei , H. Li , J. Zhang , M. Anpo , Low‐Dimensional and Nanostructured Materials and Devices, Springer, New York, NY, USA 2016, pp. 423–460.

[56] K. Skic , A. Adamczuk , A. Gryta , P. Boguta , T. Tóth , G. Jozefaciuk , Sci. Rep. 2024, 14, 30362. 39638826 10.1038/s41598-024-81030-9 PMC11621813

[57] H. O. Badr , F. Lagunas , D. E. Autrey , J. Cope , T. Kono , T. Torita , R. F. Klie , Y.‐J. Hu , M. W. Barsoum , Matter 2023, 6, 128.

[58] S. A. Ekanayake , H. Mai , D. Chen , R. A. Caruso , Chem. Sci. 2025, 16, 2980. 39840300 10.1039/d4sc04477k PMC11744683

[59] B. Ahlswede , K. Jug , J. Comput. Chem. 1999, 20, 563.

[60] B. Ahlswede , K. Jug , J. Comput. Chem. 1999, 6, 572.

[61] M. C. Zerner , Mol. Phys. 1972, 23, 963.

[62] A. Jain , S. P. Ong , G. Hautier , W. Chen , W. D. Richards , S. Dacek , S. Cholia , D. Gunter , D. Skinner , G. Ceder , K. A. Persson , APL Mater. 2013, 1, 011002.

[63] J. P. Perdew , K. Burke , M. Ernzerhof , Phys. Rev. Lett. 1996, 77, 3765. 10.1103/PhysRevLett.77.3865 10062328

[64] A. M. Koster , P. Calaminici , M. E. Casida , R. Flores‐Moreno , G. Geudtner , A. Goursot , T. Heine , A. Ipatov , F. Janetzko , J. M. del Campo , “deMon2k, version 4.4.1”, The deMon Developers, Ottawa, Canada 2010.

[65] G. Geudtner , P. Calaminici , J. Carmona‐Espindola , J. M. del Campo , V. D. Dominguez‐Soria , R. Flores‐Moreno , G. U. Gamboa , A. Goursot , A. M. Koster , J. U. Reveles , Wiley Interdiscip. Rev.: Comput. Mol. Sci. 2012, 2, 548.

[66] N. Godbout , D. R. Salahub , J. Andzelm , E. Wimmer , Can. J. Chem. 1992, 70, 560.

[67] B. I. Dunlap , J. W. D. Connolly , J. R. Sabin , J. Chem. Phys. 1979, 71, 3396.

